# Liver transplantation for NASH-related hepatocellular carcinoma versus non-NASH etiologies of hepatocellular carcinoma: A systematic review and meta-analysis

**DOI:** 10.1371/journal.pone.0317730

**Published:** 2025-03-19

**Authors:** Kunlin Chen, Ming Yang, Guangjun Li, Wentao Wang

**Affiliations:** 1 Division of Liver Surgery, Department of General Surgery, West China Hospital, Sichuan University, Chengdu, China; 2 Department of Hepatobiliary Surgery, The First Affiliated Hospital of Chongqing Medical University, Chongqing, China; University of Tsukuba, JAPAN

## Abstract

**Background:**

Non-alcoholic steatohepatitis (NASH)-associated hepatocellular carcinoma (HCC) has been emerging a predominant reason for liver transplantation (LT). The complexity of comorbidities in this population increases the possibility of poor transplant outcomes. The purpose of this study was to evaluate the differences in survival after transplantation among patients with NASH HCC and those with non-NASH HCC.

**Method:**

We conducted systematic searches of the PubMed, Embase, Web of Science, and Cochrane Library databases. To analyze the data, both fixed and random-effects models were employed to aggregate hazard ratios (HRs) along with 95% confidence intervals (CIs) for recurrence-free survival (RFS) and overall survival (OS) outcomes. This study is registered with PROSPERO as CRD42024578441.

**Results:**

A total of seven studies were included in this study. This study revealed that there was no significant difference in OS between liver transplant recipients with NASH HCC and those with non-NASH HCC. The RFS of NASH HCC patients were significantly longer. The HRs were 0.70 (95% CI: 0.51-0.97, P = 0.03) for RFS and 0.88 (95% CI: 0.72-1.07, P = 0.21) for OS, respectively.

**Conclusion:**

This study indicates that patients with NASH HCC who undergo LT have comparable OS as those with non-NASH HCC, while NASH HCC was associated with increased RFS. However, further research in randomized trials is necessary to verify these results and address potential selection biases.

## Introduction

Hepatocellular carcinoma (HCC) is one of the most prevalent and deadly cancers worldwide [1]. The epidemic of obesity has significantly contributed to the increase in cases of non-alcoholic fatty liver disease (NAFLD), which can progress to a more severe form known as non-alcoholic steatohepatitis (NASH) [2]. Consequently, the incidence of NASH HCC has increased 10-fold in the past decades [3]. Currently, there is no established treatment for NASH, which is the second most common cause of HCC necessitating liver transplantation [4]. Between 2002 and 2012, the number of liver transplants (LT) for HCC attributed to NASH in the United States increased nearly four times [5].

In recent decades, the LT field has been transformed by remarkable progress, such as the emergence of direct-acting antivirals (DAAs) [6], the enlargement of the donor pool via living donors and donation after circulatory death (DCD) donors, and the arrival of graft perfusion machines [7]. It has been over two decades since the Milan criteria was published and adopted as the global standard LT selection model for HCC patients [8]. The LT indication in HCC now features an older, more comorbid patient cohort with diminished functional reserve [9]. Despite this great import, few studies have investigated the long-term survival of patients with NASH HCC who underwent LT compared with non-NASH HCC. The subset of patients with NASH HCC, who tend to be older and have a higher body mass index (BMI) as well as a higher prevalence of diabetes and cardiovascular disease, is a complex population [10–12].

Unlike other cancer, the etiologies of liver cancer are well characterized. Thus, the differences in prognosis may appreciably change in patients when stratified liver cancers by etiologies. NASH, which involves inflammation, cell death, and fibrosis, can progress to advanced liver disease and cirrhosis, ultimately leading to the need for a liver transplant. In addition, together with cardiometabolic comorbidities, NASH is a significant contributor to both the overall and liver-associated mortality [13]. However, the effectiveness of LT for NASH HCC remains unclear. This research seeks to evaluate and contrast recurrence-free survival (RFS) and overall survival (OS) following transplantation between NASH HCC and those with non-NASH HCC.

## Results

**Fig 1:**
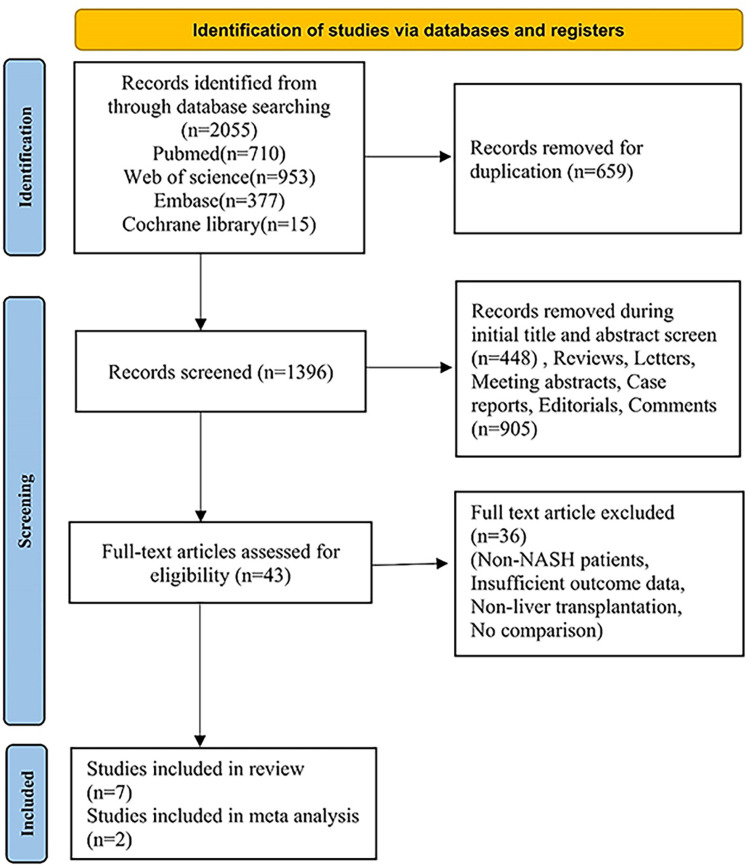
The flowchart of the study selection.

Fig 1 presents the PRISMA flow chart, which details the process of study inclusion and reasons for exclusion. Initially, a total of 2055 articles were identified through electronic searches of the PubMed (n =  710), Embase (n =  377), Web of Science (n =  953) and Cochrane Library (n =  15) databases. After removal of duplicates, there were 1396 records. After applying the inclusion criteria to the titles, abstracts and other kinds of study, 43 full-text articles were identified as potentially eligible. 36 studies were removed due to the unavailability of data [14,15].

A total of 7 articles from the United States were included in the meta-analysis [16–22]. Four study used data from the Organ Procurement and Transplantation Network (OPTN), United Network for Organ Sharing (UNOS), SRTR (Scientific Registry of Transplant Recipients) [16,18,19,22]. The OPTN, UNOS, and SRTR databases are of vital significance within the United States organ donation and transplantation framework, collaborating to guarantee equitable apportionment, efficient employment, and scientific appraisal of transplantation results. Consequently, the integration of the outcomes from the seven amalgamated studies would evidently lead to the duplication of certain patients’ data during the analysis process.

In the meta-analysis portion, we combined the results of two studies with non-overlapping temporal scopes [16,21]. Finally, a total of 12846 HCC patients underwent liver transplantation from two included studies were analysed [16,21]. The diagnoses of NASH HCC were determined using clinical information from the patient database and histologic features observed in explant pathology or preoperative biopsies in two studies [16–22]. The Newcastle Ottawa Scale scores range from 7-8, the overall quality of five study was high. Table 1 provides a detailed summary of the clinical and histopathological characteristics for all patients included in the study.

**Table 1:** Baseline characteristic of included studies.

Author		Patients	Age, year	Sex (Female)	BMI, kg/m^2^	Pre-exception MELD	Number of tumors	Median follow-up (months)
Lamm R et al	NASH	1175	64 (60–68)	378 (32.17%)	31.79 (28.20–35.53)	12 (9–16)	–	35.7 (12.7-60.3)
	non-NASH	1175	64 (60–67)	384 (32.68%)	27.75 (24.64–31.66)	12 (9–16)	–	41.4 (22.9-63.4)
Sadler EM et al	NASH	60	63.1 (60.5-67.6)	23 (38.3%)	–	11 (8-14)	1 (1-2)	56.1 (29.2-82.2)
	non-NASH	869	58.8 (54.1-62.8)	161 (18.5%)	–	10 (8-14)	1 (1-2)	–
Holzner ML et al	NASH	51	65 (63.5–67.3)	15 (29.4%)	–	15 (11–21)	1 (1–2)	54.5 (28.0–90.3)
	non-NASH	584	60 (55–65)	116 (19.9%)	–	13 (9–18)	1 (1–2)	–
Rajendran L et al	NASH	2071	64.0 (59.0–67.0)	691 (33.3%)	31.8 (28.0–35.5)	14.0 (10.0–20.0)	1 (0–1)	42.0 (39.6-45.6)
	non-NASH	18601	60.0 (55.0–64.0)	3989 (21.4%)	27.8 (24.7–31.5)	12.0 (9.0–17.0)	1 (0–1)	60.0 (58.8-61.2)
Zarrinpar A et al	NASH	33	62 ± 7.3	19 (58%)	–	25.7 ± 11.5	2.6 ± 2.5	–
	non-NASH	47	61 ± 5.9	2 (4%)	–	23.3 ± 11.7	2.7 ± 2.5	–
Zhou J et al	Group I (Donor age ≤ 34)	4723	–	1124 (23.8%)	28.55 ± 5.47	15.40 ± 8.77	1.24 ± 0.55	36.0 (18.0-60.0)
	Group II (Donor age 35–49)	3572	–	718 (20.1%)	29.00 ± 7.73	15.40 ± 8.53	1.24 ± 0.54	–
	Group III (Donor age 50–64)	3743	–	814 (21.7%)	29.04 ± 6.24	14.74 ± 7.97	1.24 ± 0.56	–
	Group IV (Donor age ≥ 65)	1238	–	329 (26.6%)	28.83 ± 5.40	14.01 ± 6.83	1.25 ± 0.55	–
Cullaro G	Female	2909	62 (57–66)	–	–	12 (8–17)	1 (0–1)	–
	Male	9802	61 (57–65)	–	–	12 (9–17)	1 (0–2)	–

NASH: Non-alcoholic steatohepatitis; BMI: body mass index; MELD: model of end stage liver disease.

### HCC recurrence after liver transplantation

Six studies reported HCC recurrence of NASH HCC after LT [16–18,20,22]. Four studies showed no difference in HCC recurrence rates between the two groups [17,20–22]. Two studies showed that diagnosis of NASH is significantly negatively correlated with HCC recurrence after LT [16,18].

### 5-year HCC recurrence after liver transplantation

**Fig 2:**
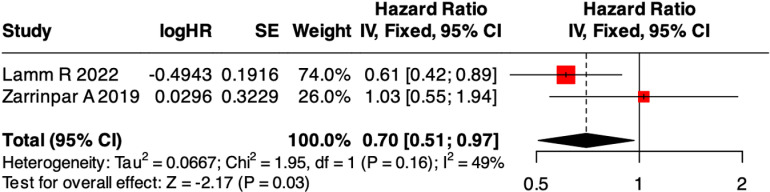
Forest plot of odds ratios for 5-year HCC recurrence after liver transplantation.

Due to overlapping time periods, potential overlapping study populations may exist among the five included studies [16–18,20,22]; thus, we chose to combine data from the two studies [16,21] with non-overlapping timeframes (2002-2012;2012-2017). A fixed effects model pooled effect sizes across studies, revealing that NASH HCC had better RFS to non-NASH HCC (HR = 0.70, 95% CI: 0.51-0.97, P =  0.03), with a moderate heterogeneity (I^2^ = 49%, P = 0.16) (Fig 2). The funnel plot analysis showed no significant asymmetry (S1A Fig).

### Overall survival after liver transplantation

Seven studies report OS of NASH HCC after liver transplants. One studies showed favorable OS in the NASH HCC group [17], while others six showed no difference in OS between NASH HCC and other etiologies of HCC [16,18–22].

### 5-year overall survival after liver transplantation

**Fig 3:**
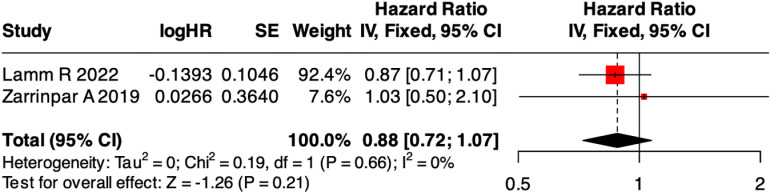
Forest plot of odds ratios for 5-year overall survival after liver transplantation.

As mentioned before, we pooled HR of 5-year OS of two studies from different time periods [16,21]. A fixed effects model was applied to pool effect sizes across these studies. Pooled analysis showed similar 5-year OS (HR = 0.88, 95%CI:0.72-1.07, P = 0.21) (Fig 3), with low heterogeneity (I^2^ = 0%, P = 0.66). No significant funnel plot asymmetry was detected (S1B Fig).

### Overall survival following post-transplant HCC recurrence

Lamm, R et al. [18]. found that OS from time of HCC recurrence was no significant difference in NASH compared to non-NASH patients. However, when assessing median time to death from recurrence in deceased patients with recurrent disease, NASH patients exhibited a notably shorter duration (150 vs. 227 days).

## Discussion

In recent years, NASH HCC has become the fastest-growing indication for LT among patients with HCC in both the United States and Europe [5,23,24]. In the current study, we compared transplant populations with NASH-related and non-NASH HCC, focusing on HCC recurrence rates and survival post-liver transplantation. Our findings show that OS are similar between NASH-related and non-NASH HCC, yet NASH HCC was associated with a decreased risk of HCC recurrence.

Obesity has reached epidemic proportions worldwide. The epidemiological trends indicate an increasing burden of disease from NASH cirrhosis complicated by HCC in the US transplant population between 2004 and 2015 [25]. With increasing control of viral hepatitis through vaccination and antiviral treatment, the prevalence of hepatitis-related HCC may decrease in the future. Furthermore, this trend contributes to fewer LT and waitlist registrations for hepatitis-related HCC [26]. A comprehensive analysis of United Network Organ Sharing (UNOS) data revealed a pivotal shift in 2016, wherein NASH emerged as the primary etiology for waitlist registrations, overtaking hepatitis infection among individuals born between 1945 and 1965 [27]. In addition, a large-scale study involving 127,164 adult patients registered for LT showed that NASH is increasingly becoming a primary reason for liver transplantation in females [28].

Individuals diagnosed with NASH were generally older and more frequently experienced conditions such as hypertension, diabetes, and cardiovascular disease. Complications arising from cardiovascular disease constitute a primary contributor to both morbidity and mortality in the adult population undergoing LT [29]. NASH-HCC patients undergoing LT often face higher risks of ineligibility due to comorbid conditions [14]. However, a research conducted by Sadler EM et al. revealed that NASH HCC showing better prognosis after LT compared to non-NASH HCC [20]. Another retrospective research utilizing the UNOS database, which included HCC patients receiving LT (comprising 1,405 NASH and 6,086 non-NASH cases), found a lower RFS in the NASH HCC group [18]. Our meta-analysis suggests that OS is comparable between patients with NASH HCC and those with non-NASH HCC following LT. However, RFS is significantly longer in patients with NASH HCC compared to those with non-NASH-related HCC

Nevertheless, the explanation for this is likely to be multifactorial. Some studies have shown that NASH HCC typically presents with fewer high-risk tumor characteristics before LT, compared to HCC from other causes. Notably, this includes a lower incidence of vascular invasion and better tumor differentiation [12,30]. Study have found that the levels of the tumor marker AFP were lower in NASH HCC compared to non-NASH HCC cases [31]. High levels of AFP correlate with poor prognosis in HCC patients [32]. However, these observations seem to be contradictory in basic research. It is well known that the common drivers of tumorigenesis modulate the microenvironment of the tumor. NASH-induced chronic inflammation suppresses cytotoxic CD8 + T lymphocytes via IgA + cells, compromising immune surveillance and facilitating HCC progression [33]. Furthermore, risk factors for NASH, including physical inactivity, insulin resistance, and lipotoxicity, persistently inflict liver damage before and after LT, which may contribute to HCC recurrence and overall survival [34]. Further in-depth studies are needed to more fully explain the observed data.

Our study has several limitations that require further discussion. First, all the included studies were from the United States. In order to avoid combining studies with overlapping populations, meta-analysis was only carried out on the data of two studies from different time periods. This might lead to the loss of some data. Second, the primary limitation of this meta-analysis is the small number of studies included in the review. Third, a significant level of heterogeneity was observed among the included studies. Forth, some studies did not control for possible confounding factors for NASH, which may influence the results significantly. Fifth, there was a lack of information to fully explore the association between beyond Milan and RFS and OS for NASH patients after LT. Sixth, all studies included in the meta-analysis were from United States, which may limit generalizability.

## Conclusions

In conclusion, our results demonstrate that patients with NASH HCC undergoing LT possess comparable OS to those of non-NASH HCC, and NASH HCC is correlated with increased RFS. However, multicenter prospective clinical trials are necessary for more accurate findings.

## Supporting information

S1 Table PRISMA 2020 checklist. (DOCX)

S2 Table List of raw analysis data. (XLSX)

S3 Table Review protocol. (XLSX)

S4 Table Newcastle-Ottawa Quality Assessment Scale. (DOCX)

S1 Fig Funnel plots for clinical outcomes. (A) 5-year HCC recurrence after liver transplantation and (B) 5-year overall survival after liver transplantation.
(TIF)
