# Supplementary material for: Liver transplantation for NASH-related hepatocellular carcinoma versus non-NASH etiologies of hepatocellular carcinoma: A systematic review and meta-analysis
Source: PLoS One. 2025 Mar 19;20(3):e0317730. doi: 10.1371/journal.pone.0317730 (PMC11922278; doi:10.1371/journal.pone.0317730)
Supplement: S4 Table — (DOCX) [file pone.0317730.s004.docx]

| Study | Representativeness of the exposed cohort | Selection of the non exposed cohort | Ascertainment of exposure | Outcome not present at start | Comparability of cohorts | Assessment of outcome | Long enough follow  up (> 3 years) | Adequacy of follow  up of cohorts | Quality score |
| --- | --- | --- | --- | --- | --- | --- | --- | --- | --- |
| Lamm (2022) | 1 | 1 | 1 | 1 | 1 | 1 | 1 | 1 | 8 |
| Zarrinpar (2019) | 1 | 1 | 1 | 1 | 0 | 1 | 1 | 1 | 7 |
| Zhou(2021) | 1 | 1 | 1 | 1 | 0 | 1 | 1 | 1 | 7 |
| Holzner(2021) | 1 | 1 | 1 | 1 | 0 | 1 | 1 | 1 | 7 |
| Rajendran(2023) | 1 | 1 | 1 | 1 | 1 | 1 | 1 | 1 | 8 |
| Cullaro(2021) | 1 | 1 | 1 | 1 | 1 | 1 | 1 | 1 | 8 |
| Sadler(2018) | 1 | 1 | 1 | 1 | 1 | 1 | 1 | 1 | 8 |
