# Supplementary figures and images for: Liver transplantation for NASH-related hepatocellular carcinoma versus non-NASH etiologies of hepatocellular carcinoma: A systematic review and meta-analysis
Source: PLoS One. 2025 Mar 19;20(3):e0317730. doi: 10.1371/journal.pone.0317730 (PMC11922278; doi:10.1371/journal.pone.0317730)

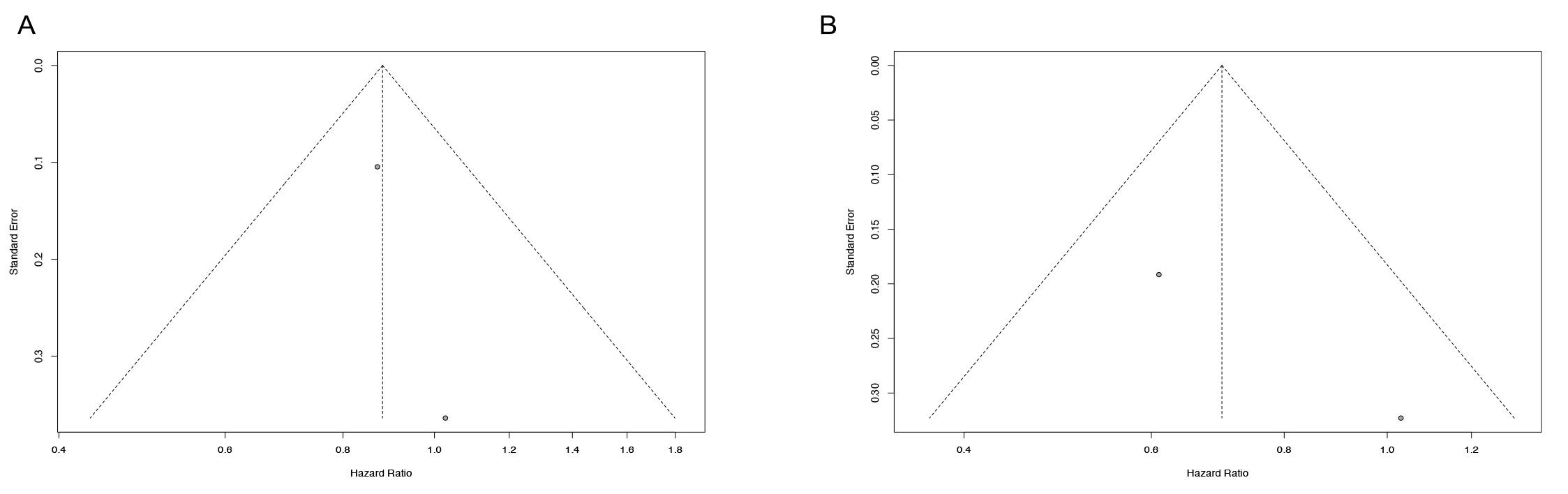

Supplement: S1 Fig — (A) 5-year HCC recurrence after liver transplantation and (B) 5-year overall survival after liver transplantation. (TIF) [file pone.0317730.s005.tif]
